# The economics of treating stroke as an acute brain attack

**DOI:** 10.1186/1741-7015-7-51

**Published:** 2009-09-23

**Authors:** Julien Bogousslavsky, Maurizio Paciaroni

**Affiliations:** 1 Center for Brain and Nervous System Disorders, Genolier Swiss Medical Network,, Clinique Valmont, 1823 Glion/Montreux, Switzerland; 2 Stroke Unit and Division of Cardiovascular Medicine, Santa Maria della Misericordia Hospital, 06126 Perugia, Italy

## Abstract

Currently, treatments for ischemic stroke focus on restoring or improving perfusion to the ischemic area using thrombolytics. The increased hospitalization costs related to thrombolysis are offset by a decrease in rehabilitation costs, for a net cost savings to the healthcare system. However, early treatment is essential. The benefit of thrombolysis is time-dependent but only a very small proportion of patients, 2%, are presently being treated with tPA. In the United States, if the proportion of all ischemic stroke patients that receive tPA were increased to 4, 6, 8, 10, 15, or 20%, the realized cost saving would be approximately $ 15, 22, 30, 37, 55, and 74 million, respectively. Being so, efforts should be made to educate the public and paramedics regarding early stroke signs. Furthermore, additional acute stroke therapy training programs need to be established for emergency departments. Finally, hospital systems need to be re-engineered to treat patients as quickly as possible in order to optimize thrombolytic benefit as well as maximize cost-effectiveness.

## Commentary

The global burden of stroke is immense [1,2] and, in fact, stroke is a major disease in both medical and economic terms. It is the leading cause of serious, long-term disability and the third leading cause of death in the USA [3]. The prevailing emphasis on cost containment and managed care has led to increased interest in the economic aspects of stroke. Even though stroke is a highly prevalent disease, effective treatment is still limited.

Currently, treatments for ischemic stroke focus on restoring or improving perfusion to the ischaemic area. The current treatment for most patients with acute ischaemic stroke is limited to the management of the symptoms, antiplatelet therapy, secondary stroke prevention and rehabilitation [4]. On 18 June 1996, the US Food and Drug Administration approved the intravenous tissue plasminogen activator (tPA) as a therapy for acute ischemic stroke within 3 hours from onset. Shortly thereafter, there was speculation that the acute costs of thrombolysis could be offset by the greater likelihood of a favourable recovery [5]. This estimation was subsequently confirmed when Markov modelling was used to demonstrate that the increased hospitalization costs were offset by a decrease in rehabilitation costs giving a net cost savings to the healthcare system [6]. It was readily observed that integrated healthcare systems (acute care, rehabilitation, and nursing home facilities) have an economic incentive to use tPA in stroke patients [7]. Other authors have supported the use of tPA as a strategy for reducing stroke costs, recognizing it as a treatment associated with important health gains (four to six quality-adjusted life-years gained per 100 patients over a lifetime) and cost savings [8-10].

Unfortunately, the enthusiasm of the late 1990s was dampened in the early 2000s with the recognition that only a very small proportion (2%) of stroke patients were actually being treated with tPA [8]. Over the past 10 years, the overall proportion of ischaemic stroke patients treated with tPA has slowly crept up and several urban and non-urban primary stroke centres report impressive proportions (10% to 20%) of stroke patients receiving tPA especially in the USA and Canada [11,12]. These findings confirm that, under ideal circumstances, a higher proportion of patients can have access to this acute therapy. Barriers to more uniform and timely access to stroke centre care and tPA continue to exist but they are being identified and addressed.

In the USA, if the proportion of all ischaemic stroke patients that receive tPA were increased to 4%, 6%, 8%, 10%, 15% or 20%, the realized cost savings would be approximately US$15, US$22, US$30, US$37, US$55 and US$74 million, respectively [13]. In Canada, the current average national tPA utilization is 1.4%. For every increase of 2 percentage points in utilization, Canadian $ 757,204 could possibly be saved annually (95% confidence interval, maximum loss of Canadian $ 3,823,992 to a maximum savings of Canadian $ 2,201,252). With a 20% rate, Canadian > $ 7.5 million could be saved national-wide during the first year [12].

However, time is crucial. In fact, treating within 3 hours of the onset of stroke symptoms is a difficult criterion to meet because the median time from stroke onset to arrival in an emergency department is between 3 and 6 hours [14]. As a result, a substantial number of ischaemic stroke patients are not eligible for intravenous tPA.

In the ECASS III trial, 800 patients received either the tPA or a placebo between 3 and 4.5 hours after the onset of symptoms. It was reported that significantly more patients had a beneficial outcome with tPA than without it [15]. However, the benefit of thrombolysis is time-dependent. In fact, tPA is nearly twice as efficacious when administered within the first 1.5 hours after the onset of a stroke compared to after 1.5 to 3 hours (odds ratio for the global outcome, 2.81 for an interval of 0 to 90 minutes, 1.55 for 91 to 180 minutes and 1.40 for 181 to 270 minutes) [16]. In comparison, in ECASS III, the odds ratio was 1.34 for an interval of 181 to 270 minutes. For one patient to have a favourable outcome (a score of 0 or 1 on the modified Rankin scale), the number needed to treat is 14 with the extended time window. This benefit is clinically meaningful and thus extends the treatment window for patients who do not arrive at the hospital early. It does not mean, however, that patients who can be treated within 3 hours should have their treatment delayed. The 'door-to-needle' time remains paramount and must be kept as short as possible in order to increase the chance of a positive outcome. Thus, all effort should be made to educate the public and paramedics about the early signs of stroke. Furthermore, additional acute stroke therapy training programmes need to be established for emergency departments. Additionally, hospital systems need to be re-engineered to treat patients as quickly as possible in order to optimize the thrombolytic benefit as well as to maximize the cost-effectiveness [17,18]. Finally, commitments need to be made to reduce the time between the onset of stroke and hospital arrival in order to be able to treat more patients with tPA. In order to achieve this, even the use of telemedicine and helicopter transport may be seen to cost-effective [19,20].

Several studies have suggested that magnetic resonance imaging (MRI) protocols using diffusion-weighted imaging (DWI) and perfusion-weighted imaging (PWI) before tPA can identify which patients may benefit from tPA within and beyond 3 hours after the onset of stroke symptoms [21,22]. Despite the increase in imaging costs and the delay in treating patients because of the increased time needed to perform MRI, penumbral-based MRI selection has shown to decrease mortality and improve functional outcome. A slight increase in total costs over a patient's lifetime due to the penumbral-based MRI selection can prove to be highly cost-effective compared to standard computed tomography-based (CT) care. In fact, the addition of penumbral-base MRI selection has been shown to increase the total cost by US$103 over the patient's remaining lifetime. However, penumbral-based MRI selection resulted in favourable outcomes more often than CT-based selection (36.66% versus 35.06%) with an incremental cost per life year of US$ 1,840 [23]. ECASS III is correct in suggesting that treatment with tPA is still effective in patients who present 3 - 4.5 hours after the onset of stroke symptoms. Furthermore, penumbral-based MRI selection of patients beyond 3 hours from onset may improve clinical outcomes. However, it must be stressed that the sooner patients arrive at hospital for tPA, the greater the health benefit and cost-effectiveness.

In conclusion, treatment with tPA is beneficial in clinical trials, effective in the real world and results in a net cost savings but only a small proportion of stroke patients are actually being treated with tPA. Research on the barriers limiting tPA use, strategies to mitigate these barriers and the experiences of advanced stroke centres suggest that increasing the use of tPA is possible. If even with small increases in the proportion of all ischaemic stroke patients who received tPA were achieved, there could an enormous savings for healthcare systems.

## Abbreviations

CT: computed tomography; DWI: diffusion-weighted imaging; MRI: magnetic resonance imaging; PWI: perfusion-weighted imaging; tPA: tissue plasminogen activator;

## Competing interests

The authors declare that they have no competing interests.

## Authors' contributions

JB and MP have participated in the manuscript and take public responsibility for the whole content. Both authors read and approved the final manuscript.

## Pre-publication history

The pre-publication history for this paper can be accessed here:


